# Responsibility, identity, and genomic sequencing: A comparison of published recommendations and patient perspectives on accepting or declining incidental findings

**DOI:** 10.1002/mgg3.485

**Published:** 2018-10-28

**Authors:** Felicity Boardman, Rachel Hale

**Affiliations:** ^1^ Warwick Medical School Coventry UK

**Keywords:** experiential knowledge, genomic sequencing, incidental findings, responsibility, UK

## Abstract

**Background:**

The use of genomic sequencing techniques is increasingly being incorporated into mainstream health care. However, there is a lack of agreement on how “incidental findings” (IFs) should be managed and a dearth of research on patient perspectives.

**Methods:**

In‐depth qualitative interviews were carried out with 31 patients undergoing genomic sequencing at a regional genetics service in England. Interviews explored decisions around IFs and were comparatively analyzed with published recommendations from the literature.

**Results:**

Thirteen participants opted to receive all IFs from their sequence, 12 accepted some and rejected others, while six participants refused all IFs. The key areas from the literature, (a) genotype/phenotype correlation, (b) seriousness of the condition, and (c) implications for biological relatives, were all significant; however, patients drew on a broader range of social and cultural information to make their decisions.

**Conclusion:**

This study highlights the range of costs and benefits for patients of receiving IFs from a genomic sequence. While largely positive views toward the dissemination of genomic data were reported, ambivalence surrounding genetic responsibility and its associated behaviors (e.g., duty to inform relatives) was reported by *both* IF decliners and accepters, suggesting a need to further explore patient perspectives on this highly complex topic area.

## 1 INTRODUCTION

The appropriate handling of “incidental findings” (IFs) is an issue that has long concerned medical practitioners (Ofri, 2013). IFs have been defined as findings that have “health or reproductive importance for an individual, discovered in the course of conducting a particular study (screening or clinical practice) but beyond the scope of that study” (Christenhusz, Devriendt, & Dierickx, 2013). From the identification of an enlarged gallbladder, to a benign brain tumor during routine investigations for other conditions, healthcare professionals in various fields of medicine frequently have to make judgments in the course of their clinical practice about whether patients should be informed of these findings given that they are unsolicited medical information, often of unclear significance, and for which prior consent to obtain them has not typically been secured.

While genetic medicine is already an area where the discovery of IFs is particularly common (Christenhusz et al., 2013), the increasing application of genomic sequencing and exploratory (as opposed to targeted) analysis techniques within mainstream NHS Healthcare has further compounded this issue. Indeed, the sheer volume of data that can be generated and analyzed through the use of genomic sequencing has been revolutionized by the emergence and increasing cost‐effectiveness of new technologies. Due to this exponential rise in available data, the potential for IFs to emerge in the context of genomic research and clinical practice has correspondingly soared, raising important ethical and social issues around the acceptability of their identification and, more pertinently, their (non)disclosure to genomic medicine patients.

While it has been widely acknowledged that the boundaries between “clinically significant” and “clinically actionable” findings within a genomic sequence are often highly uncertain or even malleable (when interpreted in the context of other relevant health data; Knoppers, Joly, Simard, & Durocher, 2006), their very generation raises significant questions around whether or not patients have the right to access them. Studies that have explored the attitudes of researchers, healthcare professionals, patients, and the general public have consistently demonstrated enthusiasm for, and interest in, receiving IFs on the parts of both the general public and genomic medicine patients, highlighting that the latter two groups harbor the most permissive views around the return of unsolicited genomic findings than any other stakeholder group (Bollinger, Scott, Dvoskin, & Kaufman, 2012; Driessnack et al., 2013; Fernandez et al., 2014; Haga, O’Daniel, Tindall, Lipkus, & Agans, 2011; Middleton et al., 2016; Ploug & Holm, 2017; Townsend et al., 2012; Yushak et al., 2016).

In the context of public and patient demand to receive them, therefore, ethical arguments both for and against the return of IFs have been extensively rehearsed in the literature in recent years (Berkman & Hull, 2014; Christenhusz et al., 2013; Gilwa & Berkman, 2013; Hens, Nys, Cassiman, & Dierickx, 2011; Hofmann, 2016; Shkedi‐Rafid, Dheensa, Crawford, Fenwick, & Lucassen, 2014). Within this literature, it has been suggested that both extreme positions in this debate (i.e., the case for full disclosure of IFs and the case for their complete nondisclosure) are ethically unacceptable (Christenhusz et al., 2013). In other words, both withholding potentially relevant health information from patients, but also indiscriminately disclosing all unsolicited findings are both viewed as both morally deplorable strategies. Indeed, the latter requires substantial (and often nonexistent) resources to be successfully implemented, whereas the former has been critiqued for its inherent paternalism and neglect of duty of care (Ravitsky & Wilfond, 2006; Townsend et al., 2013).

In order to reach an ethically sound solution to the problem of genomic IFs both in clinical practice and in sequencing research, various taxonomic systems have been developed to guide decisions around which IFs should be returned to patients and which should not (see Table 1). These taxonomies use categories or “bins” (Berg, Khoury, & Evans, 2011) to group similar IFs together in order to determine whether they should be returned to patients. While the categories used vary between studies and authors, the taxonomies generally coalesce around the following three distinct constituent components:

1. *The strength of the genotype/phenotype correlation*. This area of categorization addresses the diversity of gene penetrance and expressivity and includes IFs that relate to predispositions rather than certain genetic disease (e.g., Berg et al., 2011; Boycott et al., 2015; Klitzman et al., 2013; Leitsalu et al., 2016; Wolf et al., 2008)
2. *The impact, severity, and treatability of the associated genetic disease(s)*. This dimension of IFs appears most commonly across the taxonomies and determines the management of the IF based on the likelihood of symptoms, the age at which they will occur, their severity, as well as the degree to which the condition can be prevented or ameliorated through an intervention such as treatment or surveillance (e.g., Bennette, Gallego, Burke, Jarvik, & Veenstra, 2015; Hens et al., 2011; Himes et al., 2017; Knoppers, Deschênes, Zawati, & Tassé, 2013; Korngiebel et al., 2015; Mayer et al., 2011; Netzer, Klein, Kohlhase, & Kubisch, 2009; Sénécal et al., 2015; Van El et al., 2013).
3. *The relevance of the IF beyond the index case*. This area of categorization incorporates the rights and interests of biologically related kin to the patient, including IFs that may impact the health of existing relatives, or decisions around childbearing, for example, carrier status (e.g., Klitzman et al., 2013; Netzer et al., 2009).

**Table 1:** Taxonomies used to classify types of incidental findings to aid decision‐making about their return

Authors	Empirical or Literature‐based Study	Taxonomy
American College of Medical Genetics and Genomics (2013), American College of Medical Genetics and Genomics (2014), Green et al., (2013)	Literature	A list of 56 genes for 24 inherited disorders that should always be returned if found as an IF, regardless of the patient’s age or reasons for undergoing genomic sequencing. Amended in 2014 to allow opt‐out.
Bennette et al. (2015)	Empirical (patients’ views)	The following attributes should be evaluated in relation to any IF in order to determine whether or not it should be returned: Lifetime risk of disease Disease treatability Disease severity Whether it has reproductive implications (carrier status)
Berg et al. (2011)	Literature	IFs should be classified as follows: *Bin 1:* clearly deleterious variants with immediate clinical utility. These should be returned. *Bin 2:* variants with a known or presumed association with a disease/trait but not medically actionable. Their possible return should be discussed with the patient at the point that consent is taken. *Bin 3:* variants of unknown or no clinical significance. These should not be returned.
Boycott et al. (2015)	Literature	*Adults* Captious adults should be given the option to receive IFs (excluding those of uncertain significance or low‐penetrance dispositions). For adults lacking capacity, highly penetrant medically actionable conditions should be reported to their representative. *Children* In children, only highly penetrant actionable (during childhood) conditions should be reported to parents. Adult‐onset disorders in children should only be reported if the parents request it, or disclosure could prevent serious harm to the parent or another family member.
European Society of Human Genetics (2013), Hens et al. (2013)	Literature	Unsolicited genetic variants should only be returned if it is indicative of *a serious health problem*, either in the person tested or in their close relative(s).
Hens et al. (2011)	Literature	The following should always be returned in adults and children: *Treatable/preventable disease* All other IFs that do not fit into the above category should only be returned if requested by adult, and only once a child reaches 18 and consent renegotiated.
Himes et al. (2017)	Empirical	Carrier status IFs should only be returned if the condition to which the carrier status relates the following: *Limits the lifespan* Requires *significant medical involvement* Causes *significant physical, cognitive, or sensory impairment Demands significant daily care* and/or care cost to the family Is *risky for the mother* during pregnancy *Can be treated* to significantly mitigate or reverse symptoms
Klitzman et al. (2013)	Empirical: surveys and interviews	IFs that fall into the following eight categories should be returned: *Category 1:* high penetrance with the possibility of clinical intervention (treatment/surveillance) *Category 2:* high penetrance without the possibility of clinical intervention *Category 3:* modest penetrance with an associated clinical intervention *Category 4:* modest penetrance without an available clinical intervention *Category 5:* IFs with reproductive implications for the proband *Category 6:* IFs with reproductive implications for the children of the proband *Category 7*: pharmacogenetic variants that influence response to drugs *Category 8*: IFs that have the potential to be relevant and of interest to the proband, but have no clinical implications (e.g., paternity)
Knoppers et al. (2013)	Empirical	IFs should always be returned if the following criteria are met: *Consent:* Consent has been given by the patient *Validity:* The IF has been confirmed independently *Seriousness:* The IF poses a serious risk of a serious condition and is actionable. IFs may be considered for return if the following criteria are met: *Consent:* The patient has given consent *Validity:* The IF has been confirmed independently *Risk:* It poses an established risk to the health of the patient *Treatment:* There is therapeutic benefit to disclosure.
Korngiebel, West, and Burke (2018)	Empirical: focus groups and surveys	The following categories of IF should be returned: *Lifespan limiting (childhood):* IFs that lead to conditions where 50% of affected children die before age 10. *Serious:* default category. If the phenotype is variable, this category is used only if >50% of affected people have the most severe form. *Moderate/mild:* symptoms not life‐threatening with only mild/moderate disruption to normal activities. Treatment, if available, is not burdensome. *Unpredictable:* wide range of severity in phenotype. Factors such as age of onset and severity vary widely. *Late onset:* conditions for which symptoms do not appear until after 20.
Leitsalu (2016; Adapted from Berg et al., 2011)	Literature and surveys	*Bin 1:* medically actionable. *Bin 2a:* low risk *Bin 2b:* medium risk *Bin 2c:* high risk For Bin 1, IFs should be returned for all known/presumed deleterious gene variants. For Bins 2a, 2b, and 2c, reporting of all known/presumed deleterious gene variants should be done only if elected by the patient, through shared decision‐making with an appropriate healthcare specialist.
Mayer et al. (2011)	Empirical: case study	*Treatable diseases in children:* information that will directly impact the care of the child. *Adult‐onset treatable diseases:* information on adult‐onset disease that could be treated in adulthood. *Adult‐onset nontreatable diseases:* returned on a case‐by‐case basis.
Netzer et al. (2009)	Literature and clinical observations	The following IFs should be returned: *Predictive value:* findings that have future implications not only for the health of the proband, but also their family. *Availability of treatment:* IFs for which effective treatment options (including surveillance programs) exist. *Carrier status:* IFs that reveal carrier status for autosomal recessive disorders that may have reproductive implications for their proband and/or their family.
Sénécal et al. (2015)	Literature and consultation process	IFs concerning a child must be returned when: They meet criteria of *scientific and clinical validity* have significant health *implications effective treatment or prevention is available* and can begin during childhood/adolescence; has *approval* from parents has been *confirmed by an accredited clinical diagnostic laboratory*. IFs concerning a child should not be returned when: They concern *future health or predisposition to late‐onset disorders* or *when consent has not been received from a parent*
Wolf et al. (2008)	Literature	The following categories of IF should be returned: *Findings that offer strong net benefit:* These findings reveal conditions that are potentially grave or life‐threatening and can be treated. *Findings that offer possible net benefit:* findings that a research participant/patient may deem important, even if the condition cannot be avoided (e.g., findings related to susceptibility) The following IFs should not be returned: *Findings that have unlikely net benefit:* conditions of no clinical significance that are not life‐threatening and have no reproductive significance. This includes findings with undetermined clinical significance. These findings should not be returned.

The evidence used to support these taxonomies (Table 1), however, has largely been developed by clinicians and professional bodies, with far less data available on the way in which sequencing patients and the general public make decisions. Where the views and decisions of genomic sequencing patients and their families have been included, studies have mostly emphasized their liberal attitudes toward the dissemination of IFs, both inside and outside the clinic (Clift et al., 2015; Kaphingst et al., 2016). While there is evidence that greater ambivalence exists around IFs that relate to children (especially when the IF is not clinically actionable and/or relates to a late‐onset condition; Kleiderman et al., 2014; Sapp et al., 2014; Ziniel et al., 2014), the literature nevertheless suggests that the majority of sequencing patients overwhelmingly support the sharing of all IF information that is available to the clinician, so long as the patient requests it.

As most NHS genomic sequencing is undertaken to facilitate a diagnosis, and, as such, on people already living with unspecified long‐term health conditions, it has been argued that these groups of patients are better equipped (than members of the general public) to cope with uncertain or “bad news” results (Hitch et al., 2014), features that may characterize an IF. However, as genomic sequencing frequently relies on sequencing, not only the index case, but also other members of their (extended) family—those with less experience and knowledge of genetic disease—are also being called upon to make decisions around the return of IFs. However, the effect such contextual factors (such as prior experience with genetic disease) have on patients’ decision‐making and the reasons patients refuse receipt of genomic information have generally been under‐researched.

This paper explores this identified gap in the literature through a qualitative study of the views of people undergoing genomic sequencing as part of Genomic England’s 100,000 Genomes Project. Taking as its analytic framework the taxonomies developed by clinicians and researchers to classify and define various different types of IF (Table 1), this paper offers an in‐depth comparison of the views of 31 genomic sequencing patients (13 of whom accepted IFs and 18 of whom refused some or all IFs offered to them) with those of genetics professionals (as expressed in the literature) in order to identify areas of concordance and discordance between the perspectives and priorities of these two important stakeholder groups. By taking the patient’s perspective as a point of departure, this paper contributes to a small but emerging body of the literature designed to better understand the processes through which patients come to accept or decline IFs and, consequently, how they can be supported through this.

### 1.1 100,000 Genomes Project

The 100,000 Genomes Project is a Genomics England initiative that aims to sequence 100,000 genomes from approximately 70,000 people who are either NHS patients with a rare disease or cancer or their unaffected family members, in order to assist with obtaining a diagnosis and/or to facilitate research for their condition.

Participants in the 100,000 Genomes Project receive the results of their genomic sequence as two components: (a) the “main finding” from their genomic sequence, which concerns the health issue they came to the project with; and (b) additional findings (referred to throughout this paper as IFs) that were discovered surreptitiously during the sequence. Only variants deemed clearly pathogenic (or with a high likelihood of becoming pathogenic) and where an early intervention is both available, and deemed beneficial, are authorized for return within the project (see Table 2). These IFs are then subcategorized into two types: health‐related IFs (i.e., findings that relate to health conditions that could affect the participant and/or their biologically related kin) and reproductive IFs (findings that relate to conditions that will likely not affect the participant, but could be passed on to offspring). Participants in the 100,000 Genomes Project can choose to accept either, both, or neither of the types of IFs. They may also accept or decline individual findings within each of these two broad categories. As the list of authorized IFs is likely to expand over time, either because new genes are identified, the variant is recategorized (e.g., if a treatment becomes available), or a new category of IFs is added to the list, participants are made aware at the start of the project that they could potentially be contacted in years to come with an IF result. As such, informed consent in this context is an ongoing rather than one‐off event.

**Table 2:** Incidental Findings currently being returned by 100,000 Genomes Project

Health‐related incidental findings and variants looked for	Reproduction‐related incidental findings and variants looked for
Bowel cancer predisposition: *MLH1* (adult only), *MSH2* (adult only), *MSH6* (adult only), *MUTYH* (adult only), *APC* (adult and child)	Cystic fibrosis (CFTR)
Breast and ovarian cancer predisposition: *BRCA1* (adult only), *BRCA2* (adult only)	
Other cancer predisposition: *VHL* (adult and child), *MEN1* (adult and child), *RET* (adult and child)	
Familial hypercholesterolemia: *LDLR* (adult and child), *APOB* (adult and child), *PCSK9* (adult and child)	

There are currently six health‐related IFs on the list of approved IFs (five relating for cancer predispositions and one for familial hypercholesterolemia) with children disqualified from receiving any IF that relates to an adult‐onset condition (see Table 2). Currently, cystic fibrosis is the only reproductive IF that is being returned. Furthermore, as cystic fibrosis is inherited recessively, this finding is only returned if both members of a couple participate in the project and both agree to receive it.

## 2 METHODS

The data presented within this paper are derived from interviews with 31 patients who underwent genomic sequencing as part of the 100,000 Genomes Project at a large regional Genomic Medicine Centre in England. The data were collected between October 2017 and March 2018. These interviews were part of a larger study that compares the views of the general population taking part in genomic sequencing research with the views of individuals and families living with genetic conditions (Boardman & Hale, 2018).

Interview participants were identified through 100,000 Genomes Project clinic lists held by the regional genetics service. Participants were considered eligible if they were (a) volunteering for genomic sequencing as part of the 100 K Genomes Project, (b) over the age of 18, (c) had either accepted or declined Ifs, and (d) were able to communicate fluently in English without the need for an interpreter. Initially, genomic medicine clinic staff conducted the identification of potential participants through clinic lists and mailed out participant information sheets to 100 eligible genomic sequencing patients with a covering letter. This initial strategy of recruitment led to the successful recruitment of 22 participants, although all those who responded were IF accepters. Given that the overwhelming majority of genomic sequencing volunteers accept all IFs associated with their sequence, purposive sampling was employed to selectively target IF decliners. A second round of 40 letters were sent out, exclusively to IF decliners (including those who had declined some, but accepted other IFs), which yielded only two responses. In a final attempt to increase the number of IF decliners, follow‐up phone calls were made to each of the participants who had not responded to the letter as well as to the six decliners who had received a letter in the first round. This strategy of undertaking a follow‐up phone calls led to the successful recruitment of a further 16 IF declining participants (see Table 3).

**Table 3:** Participant characteristics and IF decisions

Participant	Accepted reproductive incidental findings?	Accepted health‐related incidental findings?	Gender	Age	Status	Condition in family
Peter	YES	YES	Male	80	Proband	Ataxia
Malcolm	YES	YES	Male	38	Proband	Cancer
Ivan	YES	YES	Male	78	Proband	Heart condition
Harvinder	YES	YES	Female	65	Proband	Heart condition
Jennifer	YES	YES	Female	31	Sister	Leopard syndrome
Roger	YES	YES	Male	59	Proband	Hereditary spastic paraparesis
Niall	YES	YES	Male	26	Proband	Friedreich’s ataxia
Emma	YES	YES	Female	40	Mother	Limb deformities
Lola	YES	YES	Female	21	Mother	Congenital ichthyosis
Jane	YES	YES	Female	51	Proband	Ataxia
Joanna	YES	YES	Female	38	Mother	Ataxia, hydratonia, hypermobility, and global developmental delay.
Ian	YES	YES	Male	38	Father	Dyspraxia and global development delay.
Abi	YES	YES	Female	44	Mother	Retinitis pigmentosa.
Heather	NO	YES	Female	65	Aunt	Aortic aneurism
Mary	NO	YES	Female	60	Proband	Heart condition
Frank	NO	YES	Male	71	Brother	Ataxia
Natalie	NO	YES	Female	41	Brother	Hereditary spastic paraparesis and multiple sclerosis
Helen	NO	YES	Female	36	Proband	Desmoid tumor and bowel cancer
Hallie	NO	YES	Female	48	Mother	Hearing loss
Nicola	NO	YES	Female	57	Mother	Cancer
Sarah	NO	YES	Female	25	Proband	Retinitis pigmentosa
Louisa	NO	YES	Female	44	Mother	Retinitis pigmentosa
Karen	NO	YES	Female	40	Mother	Mayer–Rokitansky–Küster–Hauser (MRKH) syndrome
Laura	NO	YES	Female	35	Proband	Hypermobility Ehlers Danlos syndrome
Lee	NO	YES	Male	43	Father	Retinitis pigmentosa.
Rhona	NO	NO	Female	42	Proband	Burst heart murmur, only one kidney, absent uterus, and bone structure issues
Simon	NO	NO	Male	42	Father	Ataxia, hydratonia, hypermobility, and global developmental delay.
Samantha	NO	NO	Female	42	Mother	Dyspraxia and developmental delay
Bethany	NO	NO	Female	42	Mother	Unknown degenerative disorder
Toby	NO	NO	Male	34	Proband	Muscular dystrophy
Jane	NO	NO	Female	41	Mother	Cognitive impairment

Note

Blue: accepted all incidental findings; Green: accepted some incidental findings, Orange: refused all incidental findings.

The interview schedule was developed by reference to the literature surrounding genomic sequencing, the 100,000 Genomes Project’s policy on IFs and from interviews conducted, as part of the same study, with families living with genetic diseases (Boardman & Hale, 2018). The interview schedule for this study covered participants’ experiences of, and views toward, both genomic sequencing and genetic screening, their perceptions of genomic information vis‐à‐vis other forms of health data, as well as their prior knowledge of genetic conditions, particularly cystic fibrosis, a condition for which an IF could feasibly be returned. Finally, participants were asked to recount their decision‐making around accepting or declining IFs and their anticipated uses of this information should an IF be returned to them.

Interviews were conducted via three methods, face‐to‐face interviews (*n* = 8), telephone interviews (*n* = 22), and email interviews (*n* = 1). The choice of interview method was determined primarily by the participant’s preference, ability, and health status. Face‐to‐face interviews were carried out either at the participant’s home or at the university. All interviews were transcribed verbatim (or responses collated within one document for the email interview) with names, place names, and any other identifiers removed. As such, all names reported in this paper are pseudonyms.

The data were analyzed with the help of NVivo 11 qualitative data analysis software. Open coding was conducted first to identify core themes (e.g., “stories of genomic sequencing involvement” and “meanings of genetic data”), before more specific subthemes were developed (e.g., “meaning and value of the return of carrier status as an additional finding”). A modified grounded theory approach to the analysis was used to generate new themes from the data, but also to cross‐reference the themes with the three key areas of classification that emerged from the IF taxonomies in the literature (Table 1) in order to compare professional and lay classifications of IFs. This paper presents the three core overarching themes, but also the subthemes that emerged from this analysis.

### 2.1 Editorial policies and ethical considerations

Ethical approval for the study was granted through the Health Research Authority in September 2017 (17/WM/0240 01/08/2017).

All participants in this study signed a consent form (or gave permission by email—where the participant was physically unable to write) indicating that they had been fully informed about the nature of the interview, as well as the likely uses of their data. All names and identifiers were removed during transcription of the interviews.

## 3 RESULTS

In total, 31 genomic sequencing volunteers took part in an interview, of which, 13 (42%) participants accepted both health and reproductive IFs, 12 (39%) accepted health‐related IFs but not reproductive Ifs, and six (19%) participants refused all IFs (Table 3). IF decliners are over‐represented in our sample as their perspectives are both poorly understood and under‐represented in the literature. Participants ranged in age from 21 to 80, with an average age of 46. The vast majority of the sample, 21 (68%), were women. Twenty‐eight (90%) participants were undergoing genomic sequencing due to an undiagnosed rare disease in their family, with three (10%) coming from a family affected by cancer. Thirteen participants (42%) were the “index case” in the family, that is, the person with the rare disease or cancer, meaning that the majority, 18 (58%), were unaffected family members. These family members included 11 mothers, three fathers, two brothers, one sister, and one aunt (see Table 3).

The results of the analysis are presented according to the three major themes used to classify IFs identified from the literature (Table 1).

### 3.1 The geno/phenotype correlation

The core theme of geno/phenotype correlation was a recurrent theme across the literature on the return of IFs in clinical practice and research (Table 1). While for professionals, this theme appraises IFs where the penetrance or expressivity of a genetic mutation is not clear (Klitzman et al., 2013), for sequencing volunteers, this theme emerged through their understandings and visualizations of the complex process by which a genomic finding comes to be manifested physically as a genetic disease.

In order to explore the views of sequencing volunteers on this correlation, as well as the way(s) in which it influenced decisions around accepting or refusing the return of IFs, participants were encouraged to discuss their motivations for getting involved with the 100,000 Genomes Project, their perceptions of genomic data (and the way(s) it might differ from other forms of health data), and its relationship to genetic diseases.

It was clear that from the outset that genomic data held a very particular status for participants in the project, although many found it difficult to pinpoint in exactly what ways. For some, the very difficulties associated with accessing the data and the need for specialist interpretation were part of what made the information precious and valuable, highlighting its complexity but also its invulnerability to manipulation, as Malcolm, a 38‐year‐old man and father of a young son who had joined the 100,000 Genomes Project due to cancer in his family commented:

[Genomic data]…. It’s not something you can hide from, it’s not something you can make up, it’s not something you can manipulate. Your DNA is your DNA, simple as that. So you can’t manipulate that. So to me that’s more of a pure, data more pure science than numbers that are taken from averages from surveys. This is, it’s deeper than that. It’s real, honest data. …the holy grail if you will.

Unlike other health data—such as weight and height, which fluctuate over the life course and are not unique to an individual—a person’s genome was viewed, by many participants, as an inimitable and static entity. For Malcolm, a person’s genome was the formula underpinning their human existence, the source from which all other physical and mental characteristics as well as health experiences emerged. Unlike health data, it also had social significance, forming the biological link connecting family members past, present, and future. It was this perception of his genomic data as an integral part of his personal, familial, and social identity, with the various responsibilities that be perceived as accompanying these identities that were key to Malcom’s ultimate decision to receive all IFs generated from his sequence, even those that were uncertain:

Well I think [incidental findings], I think it’s all very important. Because it gives you insight into yourself‐ what could come and bite you… it just, it gives you… it takes away some of the guess work because it gives you an educated guess to go actually this could, this follows a trend it’s being passed on…[…]… You know…. And I want to see my son grow up, I want to see him have his own family. So if it helps…. not my generation but their generation, then I’ll be happy with that, you know.……But it’s also, unless people are willing to participate fully in things like this [100,000 Genomes Project], then you’re never going to get that information…it would need to be everyone being screened…for it to then really progress. But people then would then say that’s the government wanting all your details, and all your DNA. But… idiots really. Actually, you know, it’s bigger than you. They just feel like it’s an invasion of privacy, but it’s not.

For Malcolm, his perceived responsibilities to maintain his own health, protect that of his son, but also to contribute to a wider project of genomic data accumulation that could be used to address major health problems such as cancer were all important in his decision to become fully involved with the research and to receive as much information from his genome as possible.

The intertwining of genomic data, personal identity, responsibility, and altruism was frequently mentioned drivers behind participants’ decisions to opt to receive all IFs they could, even those with reduced expressivity, with participants citing reasons such as “wishing to understand themselves,” “curiosity about who I am,” or “wanting to help others” to justify their decision to receive findings where their clinical implications were not clear‐cut. Participants also cited the possibilities of preventative treatments/lifestyle changes, screening (either self‐screening or as part of a formalized screening program), and reduced time to diagnosis as possible advantages of knowing about propensities in their genetic makeup.

For other participants, however, the uncertainty associated with IFs of variable expressivity rendered the results less meaningful and led to different understandings of responsibility. Simon was 42 years old at the time of interview and described joining the 100,000 Genomes Project because of his young daughter, Dasiy, who has ataxia, hydratonia, hypermobility, and global developmental delay of unknown origin. For Simon, his interest in the project was very specific—gaining a diagnosis for Daisy, with the associated hope of improving the management of her condition. He declined both reproductive IFs (saying that he and his wife, Jo—who was also volunteering for the project—would not have another biological child, but would instead choose to adopt) as well as health‐related IFs, which he viewed as being of limited value to his life. Simon described his decision in the following way:

So from my point of view I’m… I’ve isolated anything that can help and is to do with Daisy and that’s fine. Conditions that I may have that may come up in the future, I don’t really want to know about to be honest. It is what it is. I wouldn’t have known [if hadn’t participated in 100,000 Genomes Project], and if something came up and they went “oh, by the way, you’ve got an 80% chance‐ or whatever‐ of having cancer”, or having this, or having whatever else, will that change the way I live my life? Probably. Would it have a massive effect on my family and me? Yes. Do I want that? No. If something comes up in the future, it comes up in the future. I’d be no different as I was before it came. So yeah, no, I think, I don’t know, I think in some instances knowing something, especially when it’s not even definite…you’ve got an 80% chance of having something at some point in the future can define how you live your life and could actually destroy your life…[…]…and I have a good life….So I don’t really, I wouldn’t really want to upset it for any reason, for something may or may not happen. I don’t kind of, I don’t think like that.

Simon viewed propensities to genetic disease, rather than being part of his personal identity and sense of self as Malcolm had, as instead belonging to a particular mindset, or approach to life, which had been developed through his experiences of living with, and caring for, Daisy:

That’s the thing, you know, Daisy, you know, she’s got a condition, and it’s step‐by‐step, you deal with what comes up, and the more information that comes up, you find something else to help it, you know, and you try and progress through it. You don’t… it’s no good… it doesn’t benefit me or Daisy or Jo if we’re worrying about what’s going to happen in ten years’ time. I can’t…. I can’t enjoy what I’m doing now, but I also can’t, function and do, you know…. how are you going to deal with your day‐to‐day knowing what might happen? So yeah, not me. I wasn’t really interested in anything other than that.

While it has been suggested that people with experience of chronic health conditions are better able than those without to process and respond to uncertain and complex health information such as genetic propensities (Hitch et al., 2014; Sapp et al., 2014), like many parents of disabled children with high support needs and uncertain or life‐limiting prognoses, Simon described an approach to managing his day‐to‐day life that focused on immediate need (Heiman, 2002). Unlike Malcolm, who viewed the retrieval of as much information as possible from his sequence as an enactment of his “genetic responsibility” (Kenen, ) toward his son, for Simon, acting responsibly instead meant eschewing this information to retain a clear focus on the present. By so doing, Simon was better able to cope with, and enjoy, his current reality with Daisy, undisturbed by the potential pain of future‐orientated and uncertain health information.

### 3.2 Genetic disease severity and the return of IFs

For many participants, the acceptability of uncertain health information (such as a genetic finding of reduced penetrance) rested, at last in part, on the severity, impact, and availability of treatments for the implicated condition. This concern applied to both types of IF available through the 100,000 Genomes Project, influencing perceptions of the utility of health‐related and reproductive (carrier status) findings.

While the list of conditions for which participants could be identified as having a predisposition to, or being a carrier of, through IFs were limited to seven in the 100,000 Genomes Project (see Table 2), in describing examples of what they considered to be “serious,” participants spontaneously mentioned a range of diseases. The most commonly mentioned were cancers and heart conditions (both *n* = 6); followed by motor neuron disease (*n* = 3), cystic fibrosis, multiple sclerosis, diabetes, and blood disorders (all *n* = 2). The following conditions were also spontaneously mentioned by one participant each as an example of conditions that can be serious in their presentation: arthritis, Down’s syndrome, dyspraxia, dyslexia, asthma, cerebral palsy, dementia, lung conditions, kidney conditions, and sexual diseases. While specific conditions were listed as examples by many participants, there was a wide variety of interpretations as to what “serious” meant, and an acknowledgment that it encompassed a range of social, environmental, psychological, as well as biological factors. Due to this broad understanding of the impact of a genetic disease, participants frequently referred to different types of experience with a condition (such as “pain” or “restricted mobility”) without these necessarily being ascribed to a single diagnosis. Jennifer, for example, a 31‐year‐old woman who accepted all IFs and was participating in the project due to an undiagnosed condition in her sister described a serious condition in terms of the degree to which it affected life opportunities and independence:

Anything that would impede like a normal life physically or mentally where they couldn’t grow to be an adult and they were dependent for their whole life. I’d consider that serious if they couldn’t go to a normal school and have a normal education and be independent. So that probably covers a lot of things *[diagnoses]*.

However, for other participants, unpicking the severity of a condition from other factors, such as the likelihood of it ever developing and the social and environmental context in which the condition is experienced, was near‐impossible. While components of this information (e.g., geno/phenotype correlation) were viewed as largely objective information, however, judgments on disease severity were considered to be far more nuanced, idiosyncratic, and subjective, causing some participants to question whose role it was to make the judgment on where the boundaries around it should be drawn. Karen was 40 years old at the time of her interview, had refused reproductive IFs, and was the mother to a young daughter, Molly, who was suspected to have Mayer–Rokitansky–Küster–Hauser (MRKH) syndrome (a condition characterized by the absence of sex organs). While Karen acknowledged that disease severity was an important consideration in determining whether people should receive IFs, she called into question the authority of the medical profession to decide how severity should be defined, and therefore which results she would have the option of receiving;

…..More severe, you know, more severe kind of conditions are the ones that are going to affect… I suppose if they’re, you know, if a condition affects your life, your quality of life…[…]… although that’s different for each person……And I think, I think that’s the, there’s a line somewhere‐ so this is the threshold of things we give the information or not, but anything above this line we don’t give the information…..But I would hope not, I would definitely not agree with that. I don’t think you can ever hold back someone’s information after you’ve got that information, but I think you have to say everything above this line we need to consider that all the facts and where the benefits and detrimental effects could be for this person, before giving that information. But then who is making that decision? What right have they got to make a decision? So there needs to be a, you know, I presume a very, very strict protocol you would need to go through to make a decision on who knows what, but I wouldn’t want to be the one making those kinds of decisions!

Like Karen, many other participants also thought that the medical profession should take into account the person’s character (including their tendency toward anxiety and depression) when considering whether or not to return IFs, leading some to argue that findings related to mild conditions should not be returned at all. Natalie was 41 at the time of her interview and was participating in the 100,000 Genomes Project on account of her brother’s diagnosis with spastic paraparesis and her daughter’s diagnosis of multiple sclerosis. While Natalie opted to receive health‐related AFs from her sequence, she situated her ideas about the return of “mild” IFs and predispositions within a consumerist and commercially driven cultural milieu which she perceived as bringing with it a particularly low tolerance of risk:

I don’t know, I think you’ve got to work with the individual, you know? I think there’s probably lots of push out there for people to want to know if there’s something the matter with them, we want to control everything. And everything is serious now, no one ever says they have a headache, it’s always a migraine. And I’ve been… a lot of it is to do with finances as well, whether or not you can find these things out….whether you can get a house and get insurance, if you are right for this job, that sort of thing. And sometimes I think you can just frighten people without good cause really. So if it’s mild I really don’t think you need to know. I mean, we’ve done ok without knowing about them so far.

For participants such as Natalie, living in a risk‐adverse society which emphasizes personal responsibility for health was critical to the push toward an expanding definition of what “serious” conditions are. Indeed, while accepting health‐related IFs herself, Natalie simultaneously critiqued the rationale for providing this form of information in the first place, reflecting an ambivalence toward genomic medicine that was widespread among both IF accepters and decliners. The co‐existence of seemingly contradictory views highlights not only the complexity of responses to IFs (and their situation within broader social and cultural ideas about health and health behaviors), but also the limitations of understanding patient perspectives on genomic medicine by recourse to test acceptance or decline alone.

While the majority of participants in this study presented far more nuanced understandings of what “mild” and “serious” conditions were that incorporated broader ranges of modifying factors than those offered within the professional taxonomies, for other participants, the very concept of disease severity in relation to IF return was an entirely moot point. For these participants, using notions of seriousness or gene expressivity as a filter to determine which IFs should be returned was unacceptable, primarily because they viewed their genomic sequence as their own data, to which they should have full rights of access, irrespective of what the data meant.

Mary had just turned 60 and was being treated for a heart condition at the time of her participation in the 100,000 Genomes Project. While Mary had declined reproductive IFs (which she described as being on account of her lack of children), she described her views on IFs and her decision to receive all health‐related ones in the following way:

….you know, I think even if it’s a mild condition….it’s by the by. If somebody else knows it, then I should know it. I guess the medical profession are the people that would hold that information…But I do think that, yes, it’s an entitlement, I wouldn’t like to think somebody was keeping it from me. Or at the very least ask me if I want to know, which is what, you know, I signed the form to say, yes I would like to know please, because I don’t think they have a right to withhold my information.

For participants such as Mary, any harms of not receiving the information that had been generated from her sequence were perceived to outweigh the harms of knowing, even if they related to conditions that might be considered mild or unlikely to present. For Mary, ownership of the data was presented within a discourse of rights and entitlement and expressed as a desire to make autonomous decisions over how the data were used. For her, there was something inherently wrong with another person knowing more about her health status than she did herself, and addressing what she perceived as imbalanced access to her information overrode any of the difficulties associated with incomplete or flawed information that were raised by other participants.

The question of who owns genomic information arose in participants’ accounts not only in relation to disease severity, however, but also in discussions of participants’ rights and responsibilities to their biologically related kin, to which we now turn.

### 3.3 Incidental findings and biologically related kin

While participants described accepting health‐related and reproductive incidental findings for a host of different reasons, both future‐orientated (to assist the development of cures and treatments; to help plan their lives) and anchored in the present (enabling them to access tailored treatments and to better understand themselves), one of the most commonly mentioned reasons for accepting both health‐related and reproductive IFs concerned relationships with biologically related others. Indeed, while not specifically asked about within the interview schedule, seven participants spontaneously mentioned that they felt they had an obligation to ensure that genetic diseases did not get passed on through their family, and there was evidence of participants experiencing both shame and guilt when this had occurred. Niall, who opted to receive all IFs available, was 26 years old at the time he participated in the 100,000 Genomes Project, with a suspected diagnosis of an X‐linked (i.e., expressed in males and transmitted by females) neuromuscular condition. Niall described the impact his taking part in the project had had, both on his relationship with his mother, but also his daughter, who is likely a carrier:

….I remember phoning my mum and going, “I’ve been told about this [100,000 Genomes Project]. And she said “oh”, and one of the first things she said was “I’m sorry, I didn’t know”. And I guess she felt bad that she’d passed [undiagnosed condition] on to me, because she didn’t know. So yeah, I think people need to know what’s in their genes so they won’t have to have that conversation that me and mum had. And I said “it’s not your fault mum, I’m sorry”, and then she cried. And then I felt bad, and I felt bad that I’d passed that same burden on to my daughter. So yeah, maybe it would spare people the future pain or future problems, if they’re just open and honest, and say “look, this is what you’ve got, or you could have”, you know, people should know. Yeah, it was a tough phone call to have, and then telling my wife about it, she got really upset. And she said “well, what if we want more children?” And I remember just being positive and saying “well, it might be recessive, and we can have more children”. But if it’s something that I’m going to pass on, I’ll be honest, I don’t want them to have to go through what I go through on a daily basis. Some days are better than others and I’m perfectly fine. Other days, I don’t get out of bed because it’s just too much. Yeah. So the more people that know the better, it’s only fair.

Niall's sense of genetic responsibility, not only to his daughter, but also to his future and as yet hypothetical children, had entirely shifting since his participation in the 100,000 Genomes Project. Up until this point, Niall had not considered the potential genetic origins of his condition, nor what this information might mean for daughter, wife, and mother, as well as himself, as they considered both their future, present, and past reproductive responsibilities.

Indeed, for some participants, the perceived need to obtain, distribute, and act on genetic information within families was so powerful that those who did not co‐opt into such practices were labeled “irresponsible” or even “selfish,” as Frank, a 71‐year‐old participant commented:

….Well I think people have to think long and hard about whether they want to pass something on, and then take advice. I think it’s their job really to make sure they tell everyone who could be affected because basically you are… maybe bringing somebody into this world with a problem that you’ve got yourself, and it may even be worse, and making your life bad and their life hell…and some people are just selfish aren’t they? They don’t care if they, you know if they… if it’s going to affect somebody else. But I would say it’s your duty as a human being to look after other human beings, and certainly those within your own family, otherwise, where are we going?

While participants most frequently spoke of the need to disseminate genetic information to biologically related kin, to inform them both of their chances of developing the condition, but also their chances of passing it on, for some participants, this sense of genetic responsibility was, paradoxically, also the reason they opted to decline IFs.

Bethany was 42 at the time of her interview and had joined the project due to an undiagnosed degenerative disorder in her teenage daughter. For Bethany, it was not an absence of a sense of genetic responsibility that influenced her decision to decline all IFs, but rather her acute awareness of that accountability, and the concomitant possibility that she might be held responsible and blamed for any decisions taken if they were made in the context of genomic information:

I think that I just decided that, I thought why would you really want to know about the carrier testing? Because we just were happy to sort of get on with our life. We didn’t want to find out something that maybe there was nothing we could do about it, and then have that hanging over us for the rest of our lives, and also if you don’t know about something you can’t get blamed for it either, can you?

Like Niall and Frank, Bethany’s perception of the strong association between genetic responsibility and “genetic blame” was reflected in her views on IF decision‐making, even as these participants eventually arrived at entirely polarized decisions.

In addition to Bethany, other participants who declined IFs did not necessarily do so as a rejection of their responsibilities to biological kin, but rather because they had a broader view of those responsibilities, incorporating responsibilities to promote social justice, acceptance, and diversity in a society that views genetic impairment in typically negative ways. Toby, for example, was 34 at the time of his interview and had been diagnosed with a form of muscular dystrophy. For Toby, participation in the 100,000 Genomes Project was about gaining a definitive diagnosis and access to potentially more suitable treatments. However, he had concerns about accessing and disseminating his genomic data beyond the boundaries of this goal. Indeed, for him, declining all IFs was an active decision to demonstrate his affirmation of life with genetic impairment:

I suppose I always wonder with that [disclosure to biologically related kin] how far down the road are you going to get with that until you’re starting to verge on eugenics? Well maybe not that as such but, you know, those kind of areas….. So, you know, it’s not just affecting the person who is making the decision [about IFs], but how do you, how is that decision going to have an effect on somebody else who has got that condition, and what are you saying to them? What you’re saying to them is that, you know, you shouldn’t have been born, we want to stop you happening again so we better make sure everyone knows and does the right thing. I’m sorry, no. So yeah, that’s my, you know, I don’t like that, that idea. So, you know, people say that if the information’s available, everyone should have it, but should you be getting that information in the first place? I don’t know, but I think probably not.

Unlike Niall and Frank, Toby’s interpretation of his genetic responsibility extended beyond his biological family to other people with the same condition as him. For him, reproductive responsibility lays primarily in his reinforcement of the intrinsic value of life with a genetic disorder, rather than in the prevention of lives affected by them. Through a dislocation of his genomic data from the discourse of rights and entitlement which often surround it, Toby situated the return of IFs within a sociopolitical context in which the lives of disabled people are valued in very particular ways.

## 4 DISCUSSION

As genomic medicine continues to expand, there are mounting concerns about how the swathes of data that can be generated from its usage are accessed, stored, interpreted, and communicated to patients (Christenhusz et al., 2013; Clift et al., 2015; Himes et al., 2017; Klitzman et al., 2013). Indeed, these concerns are only set to increase as techniques such as whole genome sequencing enter mainstream health care, particularly in the fields of diagnostics and reproduction. While it is hoped that genomic sequencing will facilitate more accurate diagnoses, tailored treatments, and better information about one’s genomic health, IFs nevertheless remain a persistently controversial area, with different views in the published literature on how they should be managed (Ewuoso, 2016). In spite of this burgeoning professional literature, comparatively little is known about the views of people undergoing genomic sequencing toward the return of IFs. To the best of our knowledge, this qualitative study is the first to offer a comparative analysis between the decision‐making of geneticists, clinicians, and researchers, with the views, experiences, and decisions of 31 whole genome sequencing volunteers who had all recently made decisions about whether or not to receive them. This study is also one of the first to include the underexplored perspectives of participants who declined Ifs, a minority group within genomic sequencing patients overall, and a challenging population to recruit. However, by purposefully oversampling this group and employing more intensive recruitment strategies to do so, we were able to conduct a more in‐depth and substantial analysis of their views.

There was evidence from across the sample that genomic data were held in particularly high regard by those participating in the project and considered vastly different to other forms of health data. The need for specialist technological input to both access and interpret it, its relevance to all systems and organs within the body, but, critically, also its permanency and uniqueness, were are pivotal to the demarcation of genomic data as the “holy grail” (Malcolm) of health information. Indeed, for many participants, genomic data were regarded as “trumping” all other forms of health data—forming the very blueprint for an individual’s existence.

It was this high status assigned to genomic information by participants in the study that made the potential of an imperfect correlation between genomic findings and phenotypic expression particularly hard to reconcile. As many participants had joined the 100,000 Genomes Project with expectations of finding a “solid answer” (Hallie) to the health difficulties affecting their family, IFs that related to predispositions or that had reduced expressivity posed particular challenges to deeply entrenched beliefs about the power of genomic data. Participants typically responded to these uncertainties by drawing on fatalistic ideas about genomics in order to minimize its intrinsic uncertainties (e.g., Malcolm). While for others—particularly those who rejected IFs—probabilistic information was likened to a “sword of Damocles” hanging over them, which, if related to a condition that could not be prevented, treated, or cured, was considered to only cause anxiety and reduce enjoyment of life. This view is also reflected in the professional literature that argues for restrictions on the return of IFs (Berkman & Hull, 2014) as well as being echoed in the debates that surround the possible expansion of the newborn bloodspot screening (Taylor‐Phillips et al., 2014). Indeed, as the “therapeutic gap” (Botkin, 2016; i.e., the chasm that exists between the capacity to identity genetic diseases and the ability to treat them) appears to be widening alongside improvements in detection technologies (of which genomic sequencing is one), increasing numbers of IFs with highly uncertain impacts and few available therapeutic options are likely to continue to appear in the future, suggesting a need for ongoing regular revisions of the criteria used to determine which IFs should be returned to patients.

However, the likelihood of the genetic disease actually occurring was not the only factor that participants considered important when deciding whether or not to receive its associated IF. The severity of the condition and its anticipated trajectory were also considered to be of paramount importance, both for interview participants, as well as within published recommendations in the literature (e.g., Bennette et al., 2015; Knoppers et al., 2013; Korngiebel et al., 2015; Sénécal et al., 2015; Wolf et al., 2008; Van El et al., 2013).

Despite its significance, however, the notion of “seriousness” remains a nebulous and poorly defined concept, in relation to both whole genome and exome sequencing (Korngiebel et al., 2015; Nuffield Council on Bioethics 2018; Sapp et al., 2014), but also genomic screening (Lazarin et al., 2014; Leo et al., 2016; Molster et al., 2017), with calls for more systematic guidelines on the classification of different genetic disorders along this dimension (Ceyhan‐Bisroy et al., 2017; Crouch, 2018).

To navigate this uncertainty, participants in this study drew on a broad spectrum of lived experience with health, disease, and disability to make sense of both the IF and their decision to receive it or not (Etchegary et al., 2008). Rather than focusing on individual conditions, however, “experiential categories” were frequently used by participants as a means by which to decipher severity. Participants drew boundaries around different types of disease experience, such as “life‐limiting,” “painful,” and “treatable” to cluster groups of conditions together and define them as either serious or mild. Unlike the classifications used within the literature that have typically only examined the medical implications of a disorder (e.g., Korngiebel et al., 2015; Lazarin et al., 2014), participants’ understandings were both nuanced and broadly contextualized, incorporating social, economic, environmental, and psychological aspects of living with genetic disease. Indeed, participants not only considered the condition itself, but were also able to *personalize* that genetic risk, tailoring their appraisal of it to their unique set of circumstances and values (e.g., Simon and Daisy) and using it as a tool with which to make decisions around the return of IFs.

As well as IF accepters, IF decliners (e.g., Karen) also considered the severity of the condition associated with an IF as an important part of their decision‐making. However, this group expressed far more reticence than IF accepters about the possibility of being able to appraise the condition’s severity in advance of it occurring. As has been highlighted in critiques of IF return from the published literature (Berkman & Hull, 2014), these participants were more likely to express concerns over who has the authority to deem a condition severe (e.g., Karen), as well as to highlight the fact that definitions of seriousness are likely to alter over time, reducing the utility of an IF in predicting severe genetic disease.

A final key feature of the way in which participants described and understood their genomic information that cut across all of the three key domains explored was its tangible relationship to identity—not just personal identity and sense of self—but also to familial identity. For participants, it was the identity‐constituting nature of genomic data that led them to challenge the authority of clinicians to withhold any IFs that were generated from their sequence. By understanding IFs through a discourse of rights and entitlement, these participants discounted the relevance of professional judgments on phenotype expression and disease severity in determining access to their IFs and instead regarded their genomic data as belonging a priori to themselves. While Birch, Townsend, and Rousseau (2012) have argued that members of the public perceive geneticists as opening the lid of “Pandora's box” through genomic sequencing, the findings of this study suggest that many participants regarded geneticists as having a much less active and creative role in the generation of IFs, acting instead as the interpreter through which *pre‐existing* genomic variants could be accessed and appraised, rather than contributing to the generation or “release” of new ones.

Prior claims on the ownership of genomic data, however, not only created tensions in the relationships between patients and healthcare professionals, but was also played out in the negotiation of rights and responsibilities within families. The notion of “genetic responsibility” has been widely used within the literature to describe the range of obligations and activities undertaken by those at genetic risk (D'Agincourt‐Canning, 2001; Hallowell, 1999; Hallowell et al., 2006; Kenen, ). However, the findings of this study highlight that a broad move away from targeted genetic testing to an age‐expansive genomic sequencing brings with it new forms of “*genomic* responsibility” that goes beyond previously understood responsibilities. The most common ways that this genomic responsibility was referred to within this dataset were in relation to the perceived duty to disclose genetic information to related family members whose health could be implicated and/or to act on future‐orientated genetic risk information that could minimize the risk of disease in either their future selves or offspring. However, as this study has highlighted, participants’ sense of genomic responsibility frequently extended beyond the boundaries of their biologically related kin, reflecting an interest in the emerging project of “social genomics.” Participants such as Toby, for example, raised concerns about the directions this project may take in the future, including its impacts on the lives of disabled people. Indeed, this notion of collective responsibility for the future directions of genomics was significant even for those participants who declined IFs. For these participants, interpreting their rejection of IFs as an expression of apathy would be to underestimate the powerful discourse of genomic responsibility that they were reacting to. Indeed, the avoidance of IFs for these participants was not a rejection or disvalue of genomic information per se, but instead was a rejection of the perceived responsibilities associated with that information, for which they did not want to be “blamed” (Bethany). As such, while advances in genomic medicine are frequently justified on the basis of their extension of patient autonomy and choice, this study highlights the way that accountability to notions of genomic responsibility (personal, familial, and social) can paradoxically undermine and displace participants’ autonomy—by reducing the means available to justify and present their decision, including their right “not to know” (Berkman & Hull, 2014; Hallowell, 1999).

Overall, therefore, this study brings into critical relief the simultaneously telescopic and expansive effects that the use of genomic sequencing can have on understandings of personal and familial health, identities, and roles. By focusing on decisions around the return of IFs, this study highlights that participants’ responses to IFs were at once tightly focused (on one particular variant) but also macroscopic, taking into account their personal biographies, social and biological relationships with known and unknown others, as well as the broader sociopolitical context in which they lived. Their accounts underscore the value placed on personal choice and autonomy (and a rejection of clinical paternalism) in determining which IFs they should have access to, but simultaneously demonstrate how broad notions of genomic responsibility can have a similarly restrictive effect on IF decision‐making as those imposed by clinicians. By closing down particular ways of justifying, and accounting for decisions—particularly IF refusal—participants found themselves navigating difficult (and not previously well‐trodden) pathways, balancing the various (and sometimes competing) interests, harms, benefits, and responsibilities associated with IF return, even when this was at the expense of their own autonomy and free choice.

## 5 FURTHER RESEARCH

Further research may usefully focus on the ways in which concepts such as reproductive citizenship, genomic responsibility, and risk may be deployed to better understand the full range of responsibilities and burdens associated with participation in genomic sequencing research and clinical practice. As the capacities of genomic medicine continue to expand and consequently also the list of potential IFs that could be returned, the involvement of patient and public groups in decisions surrounding returnable variants is now of paramount importance.

The expansion of genomic medicine also challenges traditional methods of gathering informed consent from genetics patients (Lucassen, Montgomery, & Parker, 2016). Further research that explores patients’ prior experiences with health and disease, and how these relate to their perceptions of disease severity, may be particularly useful in assisting the development of patient‐orientated taxonomies of IF return that could be used to supplement existing clinical taxonomies. Such patient‐orientated taxonomies would likely include a broader range of social, cultural, and environmental factors that are currently not acknowledged in clinical taxonomies (Table 1), but which are nevertheless aspects of disease experience that can render it “severe” in the eyes of patients (e.g., the experience of social stigma and inaccessible environments). Through the generation of patient‐centered taxonomies to assist decision‐making, the process of IF return can be rendered more meaningful, particularly in contexts where participants are likely to lack any prior experience and knowledge of the condition in question.

## 6 STRENGTHS AND WEAKNESSES

This study, while representing a wide range of views and decisions, may nevertheless be biased by its reliance on 100,000 Genomes Project volunteers. As the majority of the participants in this project were having their genomes sequenced to assist, primarily, in the diagnosis of a family member (rather than for their own direct benefit), this may have contributed to accounts whereby notions of genetic responsibility were particularly emphasized. In spite of this limitation, however, the final sample demonstrated an acceptable level of diversity, with participants having a wide range of prior experiences with rare disease and cancer (see Table 3). IF decliners were also over‐represented in this study; however, the lack of prior research on their perspectives counterbalances this sampling bias as it allowed for a detailed analysis of their (difficult to access) perspectives, which is ultimately a key strength of this paper.

## CONFLICT OF INTERESTS

The authors have no conflict of interests to declare.
